# Virophages—Known and Unknown Facts

**DOI:** 10.3390/v15061321

**Published:** 2023-06-05

**Authors:** Beata Tokarz-Deptuła, Sara Chrzanowska, Natalia Gurgacz, Michał Stosik, Wiesław Deptuła

**Affiliations:** 1 Institute of Biology, University of Szczecin, 71-412 Szczecin, Poland; 2 Institute of Biological Science, Faculty of Biological Sciences, University of Zielona Góra, 65-417 Zielona Góra, Poland; 3 Institute of Veterinary Medicine, Faculty of Biological and Veterinary Sciences, Nicolaus Copernicus University of Toruń, 87-100 Toruń, Poland

**Keywords:** virophages, virophage host, virophage boost

## Abstract

The paper presents virophages, which, like their host, giant viruses, are “new” infectious agents whose role in nature, including mammalian health, is important. Virophages, along with their protozoan and algal hosts, are found in fresh inland waters and oceanic and marine waters, including thermal waters and deep-sea vents, as well as in soil, plants, and in humans and animals (ruminants). Representing “superparasitism”, almost all of the 39 described virophages (except Zamilon) interact negatively with giant viruses by affecting their replication and morphogenesis and their “adaptive immunity”. This causes them to become regulators and, at the same time, defenders of the host of giant viruses protozoa and algae, which are organisms that determine the homeostasis of the aquatic environment. They are classified in the family Lavidaviridae with two genus (Sputnikovirus, Mavirus). However, in 2023, a proposal was presented that they should form the class Maveriviricetes, with four orders and seven families. Their specific structure, including their microsatellite (SSR-Simple Sequence Repeats) and the CVV (cell—virus—virophage, or transpovirion) system described with them, as well as their function, makes them, together with the biological features of giant viruses, form the basis for discussing the existence of a fourth domain in addition to Bacteria, Archaea, and Eukaryota. The paper also presents the hypothetical possibility of using them as a vector for vaccine antigens.

## 1. Introduction

**Figure 1:**
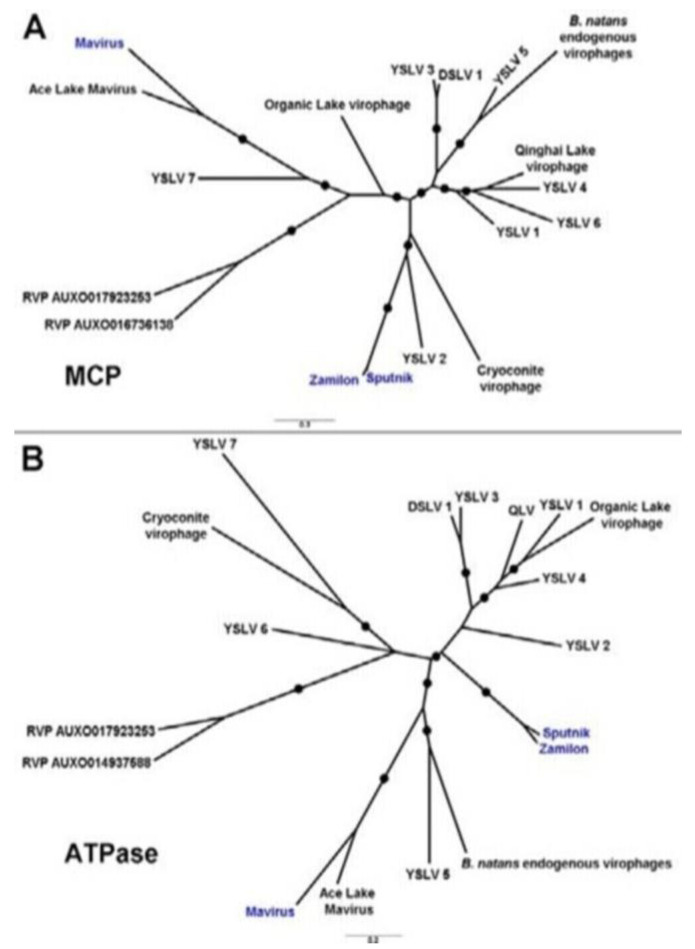
Phylogenetic relationships among virophages. Major capsid protein (**A**) and FtsK–HerA-family ATPase (**B**) sequences were aligned with PROMALS3D, and the manually edited alignment was used to create an unrooted Bayesian phylogenetic tree using MrBayes v3.1.2 with 1-million generations and a burn-in of 1000. Branches with posterior probabilities less than 0.5 were collapsed; those with posterior probabilities higher than 0.90 are marked by black dots. Cultured virophages are printed in blue [1].

Virophages, like their “host” giant viruses, are new infectious agents in the viral world. Their role in nature, mainly in the aquatic environment, but also in the context of human and livestock health, is essential [1,2,3,4,5,6,7]. It has been shown that virophages representing “superparasitism”, including almost all of those described, harm their host giant viruses [1,4,7,8]. The similarities between some virophages are shown in Figure 1 [1].

Their destructive effect on giant viruses occurs through their genetic “action”, e.g., tRNA, which influences the replication and morphogenesis of giant viruses, as well as their “adaptive immunity”, making them regulators and, at the same time, defenders of giant virus hosts, which are protozoa and algal organisms that primarily condition and shape the homeostasis of the aquatic environment [1,3,6,7,9,10]. Virophages are membrane-less viruses, having an essentially cubic-icosahedral capsid formed from their major capsid protein (MCP). They adopt a double-Galli fold, range in size from 34–74 nm (average 50–70 nm) with circular/linear dsDNA, and belong to the family *Lavidaviridae* [1,11,12,13,14,15]. However, there are now proposals [7,9] that virophages should form the class *Maveriviricetes,* with four orders and seven families. Like giant viruses and “classical” viruses, virophages are incapable of independent replication, and their process occurs in the viral particle factory of the giant viruses in which they parasitize. They can form provirophages by integrating their genomes with these viruses and host cells [1,11,12,13,14,15]. It is assumed [1,2,3,7,8,11,16,17,18] that provirophages integrate into the giant virus genome that infects protozoa and algae, as well as colonize all geographical zones because they or their genome are recorded in saltwater and freshwater environments, in terrestrial environments, and plant and animal organisms, including mammals.

Additionally, associated with the discovery of virophages is research into a three-part system termed CVV (cell–virus–virophage), which is formed by giant virus host cells, a giant virus, and a virophage, although an analogous relationship occurring between giant viruses, virophages, and transposons has also been described [1,9,18,19]. An example of such a CVV system is the giant virus, virophage, transpovirion system in amoebae (*A. polyphaga*, *A. castellani*) and flagellates (*Cafeteria roenbergensis*—now *C. burkhardei*) or the giant virus, virophage, retrotransposon system in the unicellular eukaryote *Bigelovatella natans* [8,12,18,20,21,22,23]. These recorded phenomena indicate the existence of unique commensalism in the world of these viruses, in which the transpovirion/retrotransposon uses the virophage for replication and the virophage of the giant virus, parasitizing protozoa or algae as described for the transpovirion, the Zamilon virophage, and the giant virus parasitising amoeba [19]. This recorded three-part CVV system, together with the specific structure of virophages, including their microsatellites (SSRs—simple sequence repeats), occurs 76% in their coding regions and in high density in noncoding regions. Together, with the biological features and functions of giant viruses and virophages, they provide the building blocks for the evolution of these viruses and the basis for discussing the existence of a possible fourth domain of life, which giant viruses and virophages would form after Eukaryotes, Bacteria, and Archaea [1,8,15,18,19,21,24,25,26].

An analysis of virophage genomes has shown that their dsDNA is 10–42.3 kbp long. Their genomes contain 16-to-34 genes which encode 12–39 predicted proteins that are mostly differentially expressed [11,18]. All of them have a set of conserved genes encoding the major capsid protein MCP, minor capsid protein mCP, cysteine proteinase, genome-packing ATPase, superfamily 2 helicase, and occasionally integrases, allowing them to survive as proviruses [1,15]. Protozoan host cell virophages are assumed to have a low G+C content of only approximately 30%, whereas algal host cell virophages have a high G+C content of 43–51% [15]. Different virophage protein clusters (VpPCs) have been demonstrated in virophages, allowing them to be divided into three groups [15,18]. The first includes the four previously mentioned conserved genes recorded in all virophage genomes; the second refers to typical gene families defined in 25–60% of virophage genomes but comprising only eight VpPCs (1.25%); and the third comprises 98% of all VpPCs, which was detected in fewer than 25% of all predicted virophage genomes [18]. These VpPCs of virophages may be related to their predicted function, as, for example, VpPC_007 is a site-specific adenine DNA methylase, VpPC_005 is a phage integrase/recombinase, and VpPC_012 is a phage DNA primase/helicase [18]. In addition, VpPCs of virophages, such as integrases, methylases, recombinases, and DNA polymerases, have homologues, especially in polinton and PLVs (polinton-like viruses), presumably due to gene transfers between these mobile genetic elements [18]. Of particular interest is the presence of integrases, recombinases, and tRNAs in virophages, as the integrases and recombinases identified in most of the virophage systems described presumably provide the ability to fuse their DNA with the host genome as proviruses [18].

On the other hand, the tRNAs present in virophages may serve to complement the host codon or help virophages utilize amino acids during their replication [18]. It has been shown that their presence is a result of their acquisition from the host genome, as it has been shown that tRNAs are known to be hot spots of viral integration [18], which is supported by observations that indicate that all complete virophage genomes with tRNA sequences also contain the predicted integrase gene (VpPC_005) [18]. It has been shown that despite the considerable diversity of gene content in virophages, there are also clear patterns in their genetic content specific to the group and habitat of different virophage clades [18].

Among the 39 virophages described thus far (Table 1), only 22 have been identified for the “host” giant viruses replicating on protozoa and algae, while the remaining 17 virophages have been demonstrated in metagenomic samples by identifying them using genes encoding MCP proteins [18]. These 39 virophages (Table 1) have been identified in a wide range of geographical and ecological niches; that is, in fresh inland waters, including sewage, oceanic, and marine waters, as well as thermal waters and deep-sea vents, and also in soil, plants, humans, and animals (ruminants) [18]. Genomically derived virophages from human samples have been shown to have distinct MCP protein gene models. They are likely to be associated with their lives, including food, as demonstrated, among other things, by the distribution of MCP protein gene models found in fecal samples of individuals who lived and resided in the company of baboons, cows, sheep, and arthropods [18].

## 2. Virophages with a Described ‘Host’ and Its Host Cell

### 2.1. Virophage Sputnik

**Figure 2:**
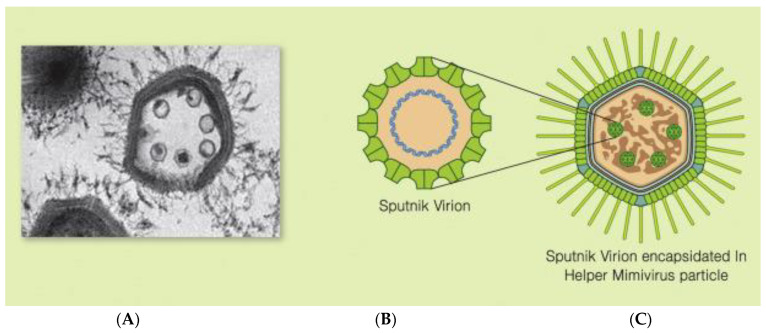
Transmission electron microscope image of the virophage Sputnik inside the Mamavirus capsid (**A**) and schematic image of the virophage Sputnik (**B**) and its location in the Mimivirus (**C**). (Wang-Shick Ryu.: Molecular Virology of Human Pathogenic Viruses, 2017. https://doi.org/10.1016/B978-0-12-800838-6.00001-1 (accessed-on 1 January 2023)—author’s modification).

During observations in 2008 by transmission electron microscopy of a viral particle factory of a giant virus, *Mamavirus ACMV* (*Acanthamoeba* (*A*) *castellani mamavirus*) resembles the giant *Mimivirus AMPV* (*Acanthamoeba polyphaga mimivirus*), which belongs to the genus *Mimivirus*, family *Mimiviridae*, and of which *ACMV* has been found in water from the Bradford cooling tower. There, small virions have been detected. They have been named after the Earth’s first satellite, the virophage Sputnik, meaning “travelling companion” [2,4,25,26,42,43] and (Figure 2).

This virophage was initially classified into satellite viruses [11,26,44,45] and is now included in the genus *Sputnikovirus* and the family *Lavidaviridae* [1,15,28]. The Sputnik virophage, similar to the other described virophages, lacks a sheath, has an icosahedral capsid 50–70 nm in diameter, and consists of 260 pseudohexameric and 12 pentameric capsomeres, which contain a circular double-stranded DNA 18,343 bp in length. This determines the V20 gene, which contains 595 amino acids, with 437 amino acids determining the AMPV giant virus MCP protein [11,24,26,42]. This indicates that the Sputnik virophage evolved from other genetic elements before associating with giant viruses [11,24,25,26,44,46]. A trimeric MCP protein is most abundant in the Sputnik virophage capsid, forming a hexagonal surface network of the molecule characterized by a triangulation number of T = 27 [26].

This MCP protein assembles pseudohexameric and pentameric capsomeres to form the outer shell of the capsid of this virophage [26]. It has also been shown that the surface of this virophage representing the pseudohexomeric capsomeres is covered by 55 Å “protrusions”, containing a triangular head protruding from the center of each pseudohexameric unit [26]. Their function is not fully known, although they are presumed to play a role in the recognition and adhesion of the Sputnik virophage to the ACMV, APMV giant virus particle, or both, allowing them and the giant virus to enter eukaryotic host cells such as amoebae [26,47]. In contrast, their capsomeres, composed of pentameric units that do not contain “protrusions”, have a type of cavity in the center of the pentamer, which can serve as a pathway for DNA exit or entry [26]. It has been shown that inside the capsid of this virophage, there is a double lipid layer 4 nm thick, which accounts for 12–24% of the lipids, in which phosphatidylserine is the main component [11,26].

The organization of the Sputnik genome is typical of viral genomes, namely a tight arrangement but little overlap of the genes. The genome virophage contains 21 genes encoding proteins ranging in size from 88 to 779 amino acids, with only slight overlap [24,25,26,44,48]. Among these are genes encoding its trimeric MCP and mCP proteins, as well as proteins predicted to be involved in its replication [24,25,26]. The genome of this virophage shows a high A+T content of 73%, which is very similar to the characteristics of the *APMV* giant virus [24,26]. Within its 21 genes, 13 have no homologues in the GOS (Global Ocean Survey) environmental databases [24]. In contrast, the remaining eight encode proteins with detectable homologues in these databases, of which three are derived from *APMV* giant virus. One is a homologue of the integrase of archeon viruses, and the other four are a predicted primase-helicase, an ATP-packing ATP-ase (homologue in bacteriophages and eukaryotic viruses), a distant homologue of the bacterial insertion sequence of the DNA-binding subunit of transposase, and the Zn ribbon protein [24,26]. Three of the proteins predicted by Sputnik, encoded by g06, g12, and g13, were most closely related to the products of the ACMV and APMV giant virus genes [24,26], except that the protein encoded by g06 is more similar to the ACMV giant virus homologue, while the proteins encoded by g12 and g13 are similar to the respective ACMV and APMV virus homologues [24,26].

Furthermore, g06 and g07 encode a protein containing a highly conserved collagen triple helix motif, while g13 encodes a protein consisting of two domains involved in viral DNA replication [24,26]. It has been shown [24,26] that the C-terminal protein domain encoded by its g13 is a highly conserved superfamily 3 helicase that clusters in phylogenetic trees with the giant virus homologue, Nucleocytoplasmic Large DNA Virus (NCLDV). In contrast, its amino-terminal portion, also encoded by g13, is a domain for which homologues with high similarity have only been detected among proteins from the GOS base set and which, due to the presence of a signature sequence motif, represents a highly derived version of the primase [24,26]. The protein encoded by g03 of this virophage has also been shown to be similar, but only to a limited extent, to the packaging ATPase of the Fts–HerA superfamily present in all NCLDVs and many bacteriophages [24,26]. Adjacent to the primase–helicase gene, g14 of this virophage encodes a protein containing a Zn ribbon protein motif and is similar to that found in several databases of proteins—GOS [24,26].

Additionally, g04 encodes a Zn ribbon protein but lacks highly conserved homologues [24,26], while the protein encoded by g10 shows significant sequence similarity to integrases from the provirus tyrosine recombinase family and archeon viruses [24]. In contrast, its protein encoded by g17 has homologues in the GOS base and belongs to the bacterial subunit/domain insertion sequence family of DNA transposase-binding transposase A proteins [24,26]. Of this virophage, g20 has been shown to encode the MCP protein, while g08 and g19 encode the mCP protein [24,26]. It has also been recorded that the closest related genes to the GOS base are its genes in the order of g13, g03, and g14, except that the protein encoded by g13 is involved in its essential replicative functions. The protein encoded by g03 is responsible for packaging its genome, and the protein encoded by g14 has a potential function in regulating its gene expression [24]. Since the FtsK-like ATPase (ATP-dependent DNA translocase) and its primase–helicase are similar to typical viral genes, the Sputnik virophage is likely related to unknown but possibly related giant viruses, which are abundantly represented in marine metagenomic sequences [24,26]. Hence, it is indicated [24,26] that Sputnik virus genes are evolutionarily related to a minimum of three distinct sources: the ACMV and APMV family of giant viruses, the family of viruses, plasmids and archeons, and another putative family of viruses.

For the Sputnik virophage, the dominant host among viruses of Lineages A, B, and C of the family *Mimiviridae* is the giant *Mamavirus* ACMV, which infects the amoebae *A. castellanii* [1,2,17,26,42,48,49]. However, with the same reaction kinetics, it can effectively infect the giant virus, *Mimivirus* APMV, of the same genus and family but living on the amoeba *A. polyphaga* [3,4,11,21,49]. It has been recorded that on the surface of these giant viruses, there are 140 nm fibrils, 1.4 nm in diameter, composed of glycosylated proteins, terminating in a protein head anchored to the capsid [26]. These fibers form a protective layer resembling bacterial peptidoglycan and are involved in the penetration of the Sputnik virophage with the giant virus into the amoebae [26]. The three proteins, R135, L829, and L725, present in *Mamavirus* ACMV filaments are essential elements [26], as the R135 protein is a GMC (glucose–methanol–choline) permeable oxidoreductase, which is probably involved in the adhesion of this virophage to *Mamavirus* ACMV, as its replication has not been detected during coinfection with its naked form [26]. The Sputnik virophage is assumed to be internalized by amoebae, ACMV, and APMV giant viruses in the same endocytic vacuole [26,28,33,46,50], and its replication occurs in the viral particle factory of these giant viruses, which harms these viruses. It has been recorded that the coinfection of Sputnik with the *ACMV* giant virus results in the formation of its defective virions, by which its replication efficiency is reduced by up to 70%. Such an effect on *ACMV* giant viruses determines its protection against amoebae [3,4,11,24,26,42,46,50]. It has been shown that after 24 h of a culture of amoebae with this giant virus, approximately 92% of these protozoa are lysed, while a coinfection of them with virophage and the giant virus results in a value of 79% [26].

### 2.2. Virophage Sputnik 2

Virophage Sputnik 2 was described in 2012 in a giant virus of the genus *Lentillevirus* of the family *Mimiviridae* that parasitized the amoeba *A. polyphaga*, which was isolated from a contact lens solution belonging to a 17-year-old patient with keratitis [11,17,25,26,43,49]. This virophage, analogous to Sputnik, has circular dsDNA genetic material, an icosahedral capsid approximately 70 nm in diameter and belongs to the genus *Sputnikovirus*, family *Lavidaviridae* [1,11,15,17,24,25,26,28,43,49]. The genome of this virophage has 18,338 bp and 20 or 21 genes, which encode proteins of 88 to 779 amino acids [11,25,26]. Its four genes are similar to those of eukaryotes and bacteriophages, three to those of APMV giant viruses, and one to archaea viruses [11,25,26]. The virophage Sputnik 2 replicates in the viral particle factory of giant viruses of the *Mimiviridae* family of Lineages A, B, and C, causing their destruction, thus showing a protective effect against their amoeba hosts cell [2,3,4,11,17,26,43,48]. With the discovery of the Sputnik 2 virophage in the giant virus *Lentillevirus*, a provirophage integrated with it was also found [12], as well as a new class of mobile genetic elements; that is, small fragments of DNA in the form of independent mobile “pieces” found in both the virophage and giant virus genomes, which have been called transpovirions [12,17,25,26,49]. These pieces are similar to the transposons (jumping genes) found in eukaryotic organisms, which can insert their DNA independently into the host cell genome or stay outside the host cell genome [25,26,42]. It is thought that, due to the presence of these mobile DNA molecules, the virophage Sputnik 2 provides a “carrier” of genes between the giant virus *Lentillevirus* and the amoeba *A. polyphaga*, which is an example of a tripartite CVV system, with a novel transpovirion forming the CVV system and consisting of the transpovirion, the virophage, and the giant virus parasitizing the amoeba [12,17,28,42].

### 2.3. Virophage Sputnik 3

This virophage was described in 2013 [4] in a soil sample collected in Marseille, France, containing *Mimiviridae* family C-lineage giant viruses. Although, as with the Guarani virophage, it has been shown that this virophage can be “free” of the giant virus [25]. This study developed a new protocol for obtaining virophages using giant viruses from the *Mimiviridae* family—Lineages A, B, and C, including *APMV* giant viruses parasitizing the amoebae *A. polyphaga* [1,2,4,5,11,17,25,26]. The virophage Sputnik 3, similar to the Sputnik and Sputnik 2, has been shown to have circular dsDNA genomes and an icosahedral capsid approximately 70 nm in diameter [11,24,43]. The genome of this virophage is similar to that of Sputnik 2. It consists of 18,338 base pairs [11,17,25,26], which are minimally smaller in number in the comparison to the 18,343 bp found in Sputnik virophage [25,43]. This virophage, analogous to the Sputnik 2 virophage, contains 20 or 21 genes, which, similar to the Sputnik virophage, encode proteins ranging in size from 88 to 779 amino acids [25,44]. Similar to the Sputnik virophage, three of the genes of this virophage are homologous to the genes of the APMV giant virus, one is homologous to the genes of archeon viruses, and four are analogous to the genes of viruses of eukaryotic organisms and bacteriophages [11,25,26]. This virophage’s remaining 12–13 genes have no detectable homologues in the GOS bases [11,26]. This mosaicism of Sputnik 3 virophage genes suggests the involvement of its genes in lateral transfers between different viruses that encode proteins of as yet unknown origin and function [25,26]. The virophage Sputnik 3, similar to the virophages Sputnik and Sputnik 2, replicates in the viral particle factory of giant viruses of the *Mimivirdae* family, mainly of Lineage C, although also of Lineages A and B, causing the formation of abnormal virions of these giant viruses and reducing their infectivity and lytic capacity against their hosts cells, which are amoebae [2,3,4,11,26,48]. Like Sputnik and Sputnik 2, this virophage belongs to the genus *Sputnikovirus*, the family *Lavidaviridae*. However, it differs from them by less than 10 base pairs, although all these virophages have a low G+C content (approximately 30%), which is also typical of giant viruses of the family *Mimiviridae* [11,15,28].

### 2.4. Virophage Sputnik Argentum

This virophage was described in 2022. Its name is derived from the giant virus *Mimivirus argentum,* on which it parasitizes and whose host is probably the amoeba *A. castellani* [28]. It is also indicated that this virophage, similar to the other Sputnik virophages, infects viruses of the *Mimiviridae* family Lineages A, B, and C [48] and is characterized by an analogous particle structure to the previous virophages [28]. Its genetic material is also a circular dsDNA, determined to be 18,800 bp, and contains as many as 26 genes and 27.93% G+C [28]. It has been recorded that 25% of the genomes of this virophage encodes five unknown or hypothetical proteins, namely, *Gp2*, *Gp5*, *Gp11*, *Gp12,* and *Gp16*. In comparison, the remaining 75% of the genome encodes proteins containing DNA-binding domains, proteins related to its morphogenesis, and proteins containing triple helix repeats, among others [28].

### 2.5. Virophage Mavirus

The Mavirus virophage was obtained in 2010 from the coastal waters of Texas, USA, from the giant virus, CroV (*Cafeteria* (*C*) *roenbergensis virus*), genus *Cafeteriavirus*, family *Mimiviridae* infecting the unicellular phototrophic marine flagellates *C. roenbergensis* [1,2,8,11,17,43,49,51]. This virophage is named for its high similarity to the self-replicating eukaryotic Maverick/Polinton transposable elements [17,52]. This virophage has an icosahedral capsid 50–60 nm in diameter, which forms only the main trimeric MCP protein and which, despite its complexity (number of triangulations T = 27), does not need auxiliary proteins when folding the capsid [17,32,42,46,51]. The genome of Mavirus virophage is a circular dsDNA of 19,063 bp. in size, presumably encoding 20 genes, among which 13 have been identified as specific genes and are g04, g05, g07–09, g10–12, and g14–18, all with a characteristic A + T content of 69.74% [8,11,17,25,32,51,53]. These genes are responsible for, among other things, coding for the main NCLD viral replication helicase, retroviral integrase, protein-primed DNA polymerase B (Polβ), endonuclease, lipase, and ATPase, as well as coding for the MCP protein and its cysteine protease [8,32,51]. The 10 genes of the Mavirus virophage have been shown to share sequence similarity with proteins of retroviruses and dsDNA viruses, as well as bacteria and eukaryotes. However, at least four proteins of this virophage, including its MCP protein, encoded by g18, are homologous to the analogous protein of the Sputnik virophage [8,11,12,17,51]. The Mavirus genome also encodes a retrovirus-type integrase and Polβ homologous to the corresponding Maverick/Polinton transposon proteins regarding gene length and content as DNA repeats and host ranges [8,12,13,41]. These genetic features of Mavirus virophage allow it to integrate at multiple sites in the CroV giant virus genome [8,13]. Studies based on DNA scoring plots of this virophage, and its phylogenetic analysis, have distinguished eight different types of endogenous virophages associated with it. The genes of these endogenous virophages are transcriptionally silent and do not undergo constitutive expression [8,13]. Thus, when an infection of *C. roenbergensis* cells with CroV giant virus co-occurs, the expression of Mavirus virophage genes is activated, leading to the replication and synthesis of its virions from proviruses [8,13]. This situation results in the *C. roenbergensis* flagellate cells transporting these provirophages not being directly protected against giant virus-CroV infection. However, when infection with this virus occurs and Mavirus provirophages are released in subsequent coinfections, they inhibit giant virus-CroV replication and protect the *C. roenbergensis* flagellates on which these viruses parasitize [13]. The protection of *C. roenbergensis* worms by proviruses against the CroV giant virus appears to take place in an altruistic model, as some cells are sacrificed to protect others [8,13]. A mutualistic relationship between the CroV giant virus and the flagellate *C. roenbergensis* has also been demonstrated, providing the Mavirus virophage with the opportunity to exist as a provirophage. In contrast, these flagellate populations benefit from the Mavirus virophage infecting CroV giant virus [13]. It is assumed [8] that the Mavirus virophage enters the CroV giant virus host cell by clathrin-dependent endocytosis or enters them independently, indicating that it enters the CroV giant virus host cell without its participation.

### 2.6. Virophage OLV (Organic Lake Virophage)

Virophage OLV was described in 2011 as an infecting agent of giant viruses of the family *Phycodnaviridae* infecting unnamed phototrophic marine algae obtained from the waters of the hypersaline Organic Lake in southeast Antarctica, whose waters have remained unchanged for decades [1,2,5,11,17,29]. The OLV virophage has an icosahedral capsid with a diameter of 50 nm [16,29], and its dsDNA genome is circular with a size of 26,421 bp, characterized by a relatively low G+C content (36.5%) [11,17,29,49,52]. The genome of this virophage probably encodes 24 genes conditioning their synthesis, 15 of which (namely, g01–11, g15, g21, g24, and g26) were identified as specific, showing 27–42% similarity in amino acids and to Sputnik virophage proteins [11,22,29]. In addition, of the proteins of the virophage OLV, six are homologous to proteins found in the virophage Sputnik [29,37], among which those found in its g20 regions encode its MCP protein, while those found in the g03 region encode a DNA ATPase. In contrast, those in the g13 region encode a putative DNA polymerase, while its homologues found in the g09, g18, g21, and g32 regions encode proteins of as yet unknown function [2,29,37,50]. The homologues of g20, g03, and g13 of Sputnik in the OLV virophage have been shown to determine its primary functions, demonstrating similarity in these virophages [29,37].

Furthermore, by studying the OLV virophage, six genes are linked to genes of giant viruses of the *Phycodnaviridae* family, indicating that gene exchange between these virophages and viruses occurs during coinfection [28,29,37]. This fact was also recorded by studying its g12, derived from an unknown giant virus infecting the alga *Chlorella sp.* [29,37]. By comparing the genome of the OLV virophage with that of the *Organic Helper Phycodnaviruses* (OLPV) of the *Phycodnaviridae* family, it was shown that as many as 7408 bp of the OLV virophage encodes g17–22 proteins, similar in 32–65% to sequences in the OLPV-1 and OLPV-2 regions of the *Phycodnaviridae* family giant viruses [29,51]. This virophage has also demonstrated unique genes targeting specific adaptation for its helper–host system, including a DNA methyltransferase specific for N6 adenine [29]. In addition, it has been described that the genes of the OLV virophage, found in g12, g13, g17, g19, g20, g22, and g23, are linked, among others, to the coding of a protein responsible for the selectivity of NCLDV viral homologues. These include APMV and Sputnik virophage, including their transmembrane protein and a protein-encoding the three-stranded structure of the OLV virophage collagen, as well as a protein presumably conditioning the interaction of the OLV virophage with the giant virus [2,29,37,50]. By infecting giant viruses of the *Phycodnaviridae* family and infecting unnamed phototrophic marine algae, the OLV virophage influences their growth and abundance, thus playing a pivotal role in regulating the microbial network of organic lake waters [29].

### 2.7. Virophage RNV (Rio Negro Virophage)

The RNV virophage was the first virophage discovered in Brazil in 2011 in the waters of the Negro River in Amazonia and was found in the amoeba *A. castellanii* infected with Samba giant virus, genus *Mimivirus*, family *Mimiviridae* Lineage A [2,8,11,17,25,30]. The capsid of the RNV virophage has icosahedral symmetry with a diameter of only 35 nm, and its genome consists of dsDNA. Although there are no precise data on its shape, it is indicated to be similar to the circular dsDNA of the Sputnik virophage [2,30]. The genome of the RNV virophage is 18,145 bp long and contains 20 genes ranging from 330 to 2340 bp, confirming its close relationship not only to the Sputnik virophage but also to the Sputnik 2 and Sputnik 3 virophages [25,30].

It is also indicated that the sequence of the MCP protein gene of the virophage RNV is also partly identical to the gene encoding the MCP protein of the virophage Sputnik [8,30]. This virophage has similar gene content and symmetry to Sputnik virophage 2, despite SNPs (single nucleotide polymorphisms) and insertions found in coding and noncoding regions [30]. A 49 bp insertion at position 11,841 in the RNV virophage has also been recorded, which results in an elongation of its region between g14 and g15 [6] and is a repeat of the previous 49 nucleotides, except for an SNP at Position 22, where cysteine has been replaced by guanine [30]. SNPs were also found in the genome of this virophage at Positions 16.075 and 18.121, with a deletion at Position 18.145 and an additional three guanines inserted at Position 18.016 [30]. Comparing the genome of the RNV virophage with that of the Sputnik, Sputnik 2, and Sputnik 3 virophages, it can be observed that it lacks the last 244 bp [30]. This virophage replicates in the Samba giant virus infecting *A. castellanii* amoebae, causing its defective shape and abnormal capsid and reducing the abundance of this giant virus in amoebae by more than 80% [2].

### 2.8. Virophage PGVV (Phaeocystis Globosa Virus Virophage)

The PGVV virophage was obtained in 2013 from the giant virus PgV–16T (*Phaeocystis globosa virus*), genus *Prymneovirus*, family *Phycodnaviridae*, infecting algae of the genus *Phaeocystis* residing in the Dutch coastal waters of the North Sea [1,2,17,21,31]. At the time of discovery, it was considered to be most closely related to Mavirus virophage and OLV. However, identification in PLV metagenomic datasets showed that it was not a virophage but a PLV [1,3,21,31]. It was justified by the lack of a cysteine protease conserved for virophages and a distinct version of the genes for MCP, mCP, and ATPase in it, as well as PLV viruses [1,21,31]. Its circular genome is a double-stranded DNA of 19,527 bp in length, containing only 36% G+C, housed in a capsid of icosahedral symmetry and 50–80 nm in diameter, which encodes 16 predicted genes, three of which show similarity to genes located in OLV and Mavirus virophages [1,2,15,21,31,40]. The PGVV virophage replicates as a linear plasmid in the viral particle factories of the PgV-16T giant virus, or as a provirus integrated into this giant virus, occurring at multiple sites in its genome [1,2,21,31,40]. It is understood [21] that the genome of the PGVV virophage associated with the PgV–16T giant virus has only three homologous genes, including primase, and cannot exist as a free viral particle, which probably represents the first example of a mobile virophage element; that is, a transpovirion. Initially, it was thought that the PGVV virophage had no recognizable genes coding for its capsid proteins. However, it has now been shown that its g12 region probably encodes a distant version of the double-gallate MCP protein, and g10 encodes a minor mCP capsid protein, which would support the theory that it is a virophage and not a PLV [1,2,21,31]. It should be added that the PLV viruses described in 2023 coinfecting the alga *Phaeocystis globosa*—14T (PgV-14T), together with the giant virus *Phaeocystis globose,* are probably new virophages that have been named PLV “Gezel–14T”, which are different from all known virophages but also have a destructive effect on specific giant viruses [6].

### 2.9. Virophage ALM (Ace Lake Mavirus)

Virophage ALM was described in 2013, identifying it as one almost complete gene sequence composed of short metagenomic reads obtained from the waters of Antarctic Lake Ace [1,2,11,17,22,25,32]. This virophage infects giant viruses, probably of the family *Mimiviridae* replicating in unspecified protozoa [2,22]. The symmetry of the capsid of this virophage has not been described, while its circular double-stranded DNA is 17,767 bp long, contains 26,7% G+C, and encodes 22 genes, including 14 homologous to the genes of Mavirus virophage, among other genes for Polβ that determine its unique evolutionary traits. Hence, it was named ALM virophage [11,22,25]. Although the genome sequence of the ALM virophage is not entirely known, it is accepted that its genome provided the first insight into sequence diversity in the Mavirus virophage subgroup [1,22]. This virophage and the Mavirus virophage have 20 and 13 of the 22 predicted genes, respectively, the sequence of which is conserved, but up to seven have undergone inversions [1,22]. The ALM virophage also shows similarity to four metagenomic YSLV virophages 1–4, with the highest found against YSLV virophage 3 and the lowest against YSLV virophage 4 [22].

### 2.10. Virophages YSLV 1–4 (Yellowstone Lake Virophages 1–4)

These virophages were identified in 2013 as four metagenomic sequences in Yellowstone Lake, USA, which were named virophages YSLV 1–4 and probably infected viruses of the family *Phycodnaviridae,* parasitizing unnamed algae or giant viruses of the family *Mimiviridae* colonizing amoebae [1,2,17,22,32,37]. Their genetic material is dsDNA, and their genomes are 23–30 kbp in size and arranged in a capsid of indeterminate symmetry and diameter. Although based on phylogenetic reconstruction, they have been shown to belong to different subgroups of virophages [1,22,32,37] (Figure 1). Most of the discovered YSLV virophage sequences 1–4 were homologous to the virophage OLV infecting giant viruses of the family *Phycodnaviridae*, which infect unnamed algae, and the virophages ALM, Mavirus, and Sputnik, which infect giant viruses parasitizing protozoa [2,22,32,37]. The dsDNAs of virophage YSLV 1–4 have been shown to range from 23,184 (YLSV2) to 28,306 bp (YSLV 4) in length, have a G+C content of 33.4% (YSLV 1) to 37.2% (YSLV 4), and encode 21 (YSLV 2) to 34 (YSLV 4) ORFs [22].

### 2.11. Virophage Zamilon

The virophage Zamilon was described in 2014 in soil samples collected in Tunisia, in which a Mont1 giant virus belonging to the family *Mimiviridae* was found to infect the amoebae *A. polyphaga* [1,2,11,17,25,33]. The name of this virophage in Arabic is “xamilon”, which means neighbour and is linked to the fact that this virophage, unlike other known virophages, does not affect giant viruses, as it does not produce their morphologically abnormal virions and does not affect their lysis [2,11,25,33]. The Zamilon virophage replicates in the viral particle factories of giant viruses of the *Mimiviridae* family of Lineages C and B but not A [25,33]. Its capsid has a helical symmetry of approximately 60 nm in diameter [16,17,43,46], and its circular dsDNA genome consists of 17,276 bp with a G+C content of 29.7% and contains 20 genes ranging from 222 to 2337 bp in length [11,17,33]. Approximately 6000 bp from the end of its genome contains an inverted portion, also recorded in giant viruses of *Mimiviridae* Lineage A [33]. The genome of this virophage is as much as 75% identical to that of Sputnik virophage and has 76% nucleotide identity, resulting in the vast majority of its genes showing high similarity with those of Sputnik virophage [11,25,36]. Its 17 out of 20 genes show homology with Sputnik’s gene with an identity of 31–86%, of which two genes show an additional 50–67% similarity with *Megavirus chiliensis* giant virus of the family *Mimiviridae*. One of its genes is 72% identical to the gene of *Moumouvirus monve* giant virus of the family *Mimiviridae* [3,11,33,47]. Of this virophage, g12 is the closest homologue of the V9 Sputnik gene, which encodes an unidentified protein but is also related to the putative cysteine protease protein of Mavirus virophage, as it has 32% identity and 83% similarity [33]. Additionally, g11 and g18 of the virophage Zamilon are closely related to the gene of the virophage Sputnik, encoding a potential integrase and a DNA-packaging protein with a putative ATPase domain [33]. The more significant homology of g19 of this virophage, with giant viruses of the *Mimiviridae* family Lineages B and C more than with Lineage A and the virophage Sputnik, has been shown to determine its infectivity [25]. It has also been demonstrated that its g08 is a homologue of Sputnik’s g14, which, as in the Zamilon virophage, has no predictable function [33]. Of this virophage, g01 and g02, showing some similarity to g15 and g02 of Sputnik (≥30% identity), is a predicted protein sequence encoded by g01, which contains a putative protein domain related to the transmembrane domain of Cytochrome C oxidase Subunit II [33]. It was also recorded that the protein sequence encoded by Zamilon virophage g09, which encodes a putative helicase, shows homology to the putative DNA primase/polymerase of virophage OLV and the putative DNA primase of virophage PGVV [33]. The virophage Zamilon also exhibits a unique evolutionary feature: the ZnR ribbon protein domain [11]. It is assumed that the functions of the proteins encoded by its gene, homologous to those of the Sputnik virophage, are probably transposase but also proteins that determine the formation of its capsid [33]. It has also been indicated that the virophage Zamilon shares a common trimeric fold with noumea viruses for its receptor-binding proteins, which may also be responsible for the host cell receptor for the giant virus [54].

### 2.12. Virophage RVP (Rumen Virophage)

Various metagenomes, including those from the activated sludge of a freshwater seawater and wastewater bioreactor and the rumen of sheep, were described in 2015. Sixteen virophage sequences carried the MCP protein in the capsid [1,2,17,25,27,55]. Two nearly complete and two partial genomes of these virophages, collected from the sheep rumen metagenome, were named rumen virophages (RVPs) [25,27,55]. The genome of the RPV virophages is linear as polintons, with the longest polinton being 26,209 bp long and encoding 22 genes [1,25,55]. Of these 22 genes, the three relatively longest ones presumably encode a protein similar to the Polβ subunit of various polintons, while the others encode “unspecified” proteins described in the GOS database, in addition to a polynucleotide kinase [25,55]. The RVP virophage most likely infects giant viruses of the family *Mimiviridae*, replicating in unspecified eukaryotic protist hosts cells living in the rumen of sheep [2,17,25,55]. Their capsid has no described symmetry, and no integrases were detected in the genome of these virophages, which may suggest that they parasitize giant viruses without integration into their genome. However, their occasional integration via an in-trans integrase cannot be excluded [55]. RVP virophages may also be hybrids of virophages and polintons capable of forming infectious virions with genes of MCP, ATP-ase, cysteine proteinase, and self-replicating eukaryotic Polinton/Mavericks transposable elements that encode Polβ together with protein primers [1,25,55]. To date, the minor mCP protein has not been found in the genome of the RVP virophage, indicating that the construction of its capsid differs from that characteristic found in many virophages, including Sputnik and Mavirus virophages [1,25,55].

### 2.13. Virophage DSLV1 (Dishui Lake Virophage 1)

In 2016, metagenetic material later defined as a virophage, which was named DSLV1 (Dishui Lake Virophage 1), was obtained from the waters of artificial Dishui Lake in Shanghai, China; it has also been found in other freshwater bodies [1,2,17,25,34,40,56]. It is assumed that although no giant viruses have been attributed to this virophage, they are likely to be giant viruses of the family *Phycodnaviridae*, which are thought to infect unspecified freshwater algae [2,34]. Virophage DSLV1 has indeterminate capsid symmetry, and its genome is a spherical double-stranded DNA with 43.2% G+C [50]. Metagenomic analysis has shown that it is 28,788 bp long and contains 28 genes, 15 of which show homology to genes of described virophages, particularly those identified in Yellowstone Lake; that is, virophage YSLV. However, two genes of this virophage are similar to giant viruses of the family *Phycodnaviridae* [11,34,51,52]. It is also indicated that more than half of the 28 genes of the DSLV1 virophage have the highest sequence similarity to the genes of the YSLV 3 virophage (33–70%) [34]. Among these, five genes are genes encoding MCP protein, mCP protein, DNA helicase, packaging ATPase, and cysteine protease [34]. In addition, the DSLV 1 virophage genome examination revealed five highly conserved regions shared between DSLV1 and YSLV 3 virophages, suggesting that the two virophages are related [34]. It has been reported that in samples in which DSLV1 virophages were found, 46 other virophage sequences were recorded, including six MCP protein-related genes closely related to OLV and YSLV virophages—mainly YSLV 3, where similarity was determined to be 33–70% [25].

### 2.14. Virophage QLV (Qinghai Lake Virophage)

This virophage was identified in 2016 in the surface waters of Qinghai Lake in the mountains of Tibet, which is rich in a planktonic microbial community [1,2,33,35]. This virophage has been shown to possibly replicate in giant viruses of the family *Phycodnaviridae*, parasitizing unnamed freshwater algae [35], and is most closely related to the virophage OLV and the virophages YSLV 1–7, particularly YSLV 1–4 [1,2,35]. The QLV virophage has an undefined capsid, and its genome is a circular dsDNA of 23,379 bp in length, with a G+C content of only 33.2%, forming 25 genes [35]. An analysis of its gene content has identified genes considered to be universally conserved for both QLV and other virophages, including genes encoding the FtsK–HerA family ATPase (g01), cysteine protease (g06), MCP protein (g18), mCP protein (*g19*), and DNA helicase/primase/polymerase (g23) [35]. The products of its core genes have also been shown to be responsible for the replication of its DNA and the packaging of its virions [35]. This virophage has seven gene homologues coinciding with the YSLV3 virophage (41% amino acid identity), eight with the OLV virophage (39% amino acid identity), nine with Virophage YSLV1 (40% amino acid identity), and 11 with Virophage YSLV4 (46% amino acid identity). In addition, its amino acid identity with Virophages Sputnik, Mavirus, Zamilon, and ALM is determined to be less than 35% [29,35]. In addition, it has been shown that its genes, g02 and g19, successively encode a glycoprotein and RecB family recombinase-containing protein, which is a subunit of the RecBCD enzyme that rescues recombinant DNA repair and causes double breaks in the DNA strand [35]. This protein also affects its glycoproteins in forming its capsid and is vital in adhesion processes and interactions between the virophage, the giant virus, and its host cell [35]. It was also indicated that the amino acid sequence of the gene encoding Gp02 of the QLV virophage has less than 48% amino acid identity to *Phycodnaviruses* (*Paramecium bursaria*, *Acanthocystis turfacea* and *Chlorella virus*), which are known to infect unicellular green algae [35]. Of its 25 genes, 11 are specific, as they have not been found in other virophages [29,35], and it is further indicated that its evolutionary affinity with OLV-like virophages and the homology of its genes, especially g02, with giant viruses of the family *Phycodnaviridae* is evidence that it replicates in these viruses [35,49].

### 2.15. Virophage Plantowirus Saccamoebe “Comedo”

This virophage was found in 2018 in the giant virus KSLT-5, probably belonging to the genus *Mimivirus*, family *Mimiviridae*, which parasitizes the amoeba *Saccamoeba lacustris*, which lives in sycamore trees [5,25]. This virophage has an icosahedral capsid 50–60 nm in diameter, but its genome (probably DNA), amount of G+C and number of genes have not been described [5,25]. When infecting the KSL-5 giant virus with this virophage, it has been recorded to affect the formation of its defective particles, demonstrating its protective effect against amoebae that are infected with KSLT-5 giant virus. Hence, it has been suggested to call this virophage “comedo”, from the Latin word *comedere*—to eat, devour [5,25].

### 2.16. Virophages CpV–PLV Curly, CpV–PLV Moe, CpV–PLV Larry

These three virophages (CpV-PLV Curly, CpV-PLV Moe, and CpV-PLV Larry) were described in 2019 along with the CpV–BQ2 giant virus from the family *Phycodnaviridae*, which infects the freshwater alga *Chrysochromulina parva*, living in the waters of Lake Tai in China and Lake Erie in the United States [25,36]. Initially, no particles were recorded during the isolation of the giant virus CpV–BQ2, the host of these virophages, as it is likely that particles of this virophage, or its genome, were packed into the giant virus, analogous to that of the virophage PGVV and the giant virus PgV–16T [36]. Further studies [36] found the genomes of these virophages and showed that they encode, among other things, the major capsid protein MCP and the minor capsid protein mCP, although it is still not described whether these three virophages are provirophages or whether they are virophages that remain in the CpV-BQ2 giant virus [36]. It has only been accepted that they belong to the PLV group of viruses, possessing between 19 and 23 genes, including all the core genes of PLVs and several genes involved in modifying their genome [36]. To date, the symmetry and size of their capsid have not been determined, and their genome is dsDNA [36]. The CpV–PLV Curly genome’s length was determined to be 22,761 bp, and its G+C content was only 37.8% [36]. Its genome encodes 19 genes, eight of which have predicted functions, as g11 of this virophage has been shown to encode the mCP protein, g12 the MCP protein, and g17 encodes a hypothetical protein, similar to that encoded by the homologous gene of the QLV virophage [36]. This virophage is closely related to the PGVV, YSLV 1, and YSLV 3 [36]. In addition to the MCP and mCP proteins, and the packaging ATPase, helicase superfamily 3 and tyrosine recombinase, it also has five uncharacterized conserved proteins common only to YSLV virophages [36]. The CpV–PLV curly virophage also encodes, in addition to genes typical of virophages, a probable endonuclease populating with the HNH (helix–turn–helix) structural motif and a DNA methyltransferase [36], and in addition, its one gene probably encodes an E3 ubiquitin ligase [36].

In contrast, the CpV–PLV Moe virophage was shown to be very similar to the CpV–PLV curly virophage and, despite having a genome of 21,750 bp with only 30.1% G+C content, encodes 23 genes [36]. Meanwhile, in the case of the CpV–PLV virophage Larry, its genome was the largest among these three characterized virophages at 22,879 bp, with 39.3% G+C content, but it encoded only 20 genes. It has the same core elements as CpV–PLV Curly and CpV–PLV Moe virophage, albeit with the 5′ half of the genome inverted [36] and, unlike CpV–PLV Curly and CpV–PLV Moe virophage, it probably encodes a DNA cytosine methyltransferase [36].

### 2.17. Virophage CVV–SW01 (Chlorella Virus Virophage)

In 2022, a *Chlorella* virophage, CVV–SW01, residing in the giant *Chlorella* virus XW01 (CV-XW01) of the family *Mimiviridae*, which parasitizes algae of the genus *Chlorella*, was obtained from the waters of Lake Dishui [23]. Describing this virophage, the CVV system was discovered in these organisms, which are unicellular eukaryotic hosts [23]; this has been recorded in the protozoa and unicellular eukaryote *Bigelovatella natans* [8,12,18,20,21,22,23]. These facts led to studying the CVV system as a potential mechanism influencing ecological phenomena in aquatic environments, including the evolution of giant viruses and virophages [23]. It has been recorded that this virophage has icosahedral symmetry of the capsid, and its circular dsDNA genome is 24,744 bp and contains only 35.6% G+C. Its genome encodes 23 genes, 13 of which have homologues in the virophage DSLV5, indicating their close affinity [23]. The genome of the virophage CVV–SW01 encodes conserved genes for these microorganisms; that is, the packaging ATPase, cysteine protease, MCP and mCP, and one of its genes probably encodes a DNA helicase [23]. This virophage is closely related to Lake Dishui virophages, particularly Virophage DSLV5; it also shows an affinity for Lake Mendota virophages and YSLV 3 [23].

Furthermore, as many as 82 genes of this virophage’s CV–XW01 giant virus host show homology with the CroV giant virus, which is most closely related [23]. It should be added that the codon usage preferences of the giant virus CV–XW01 and the virophage CVV–SW01 are very similar to those of the giant virus CroV and its virophage Mavirus, respectively, suggesting that the giant virus CV–XW01 hosts the virophage CVV–SW01 [23]. Furthermore, the giant viruses CV–XW01 and CroV show a 74.7% genomic sequence identity, indicating that the giant virus CV–XW01 may be the second species of the genus *Cafeteria* or the first species of a new genus closely related to it. It should be added that despite the close relationship between the two giant viruses, CV–XW01 and CroV, their virophages are poorly related. Given these facts, it is suggested that the interaction of the virophage CVV–SW01, the giant virus CV–XW01, and the alga *Chlorella* sp. is likely to be different from the interaction of the virophage Mavirus—giant virus CroV—the flagellate *C. roenbergensis* (now *C. burkhardaei*) [23]. Notably, Dishui Lake virophages, the closest relatives of virophage CVV–SW01, are likely to parasitize the Dishui Lake 1 green algal giant virus, which is poorly related to the giant virus CV–XW01 [23]. It has also been reported that there is evidence of interspecies infections by virophages, which may be because virophages, through horizontal gene transfer and recombination, are “linked” to a dynamic network integrating mobile genetic elements, such as the Maverick/Polinton transposon, PLVs, proviruses, transpovirons, or retrotransposons, and thus can acquire versatile adaptations to colonize and parasitize different giant viruses [23].

## 3. Virophages with an Undescribed or Probable ‘Host’ and Their Possible Host Cell

### 3.1. Virophages YSLV 5–7 (Yellowstone Lake Virophages 5–7)

The virophages YSLV 5, YSLV 6 and YSLV 7 were identified by metagenomic analyses in 2013 in water samples from Yellowstone Lake, USA, without identifying a giant virus for them and their host cell; they share genetic homology with the virophage Zamilon [1,2,3,17,37]. The symmetry of the capsid of these virophages has not been determined, but it has been shown that their genome consists of a circular dsDNA [2,37], which in virophage YSLV 5 is 29,767 bp and 32 genes, Virophage YSLV 6 24,837 bp and 29 genes and virophage YSLV 7—23,193 bp and 26 genes [45]. Phylogenetic analysis showed that they probably belong to different subgroups, as Virophage YSLV7 represents a new fourth lineage of these virophages and, together with Virophage YSLV5, is unrelated to Virophage YSLV 6 and Virophage YSLV1–4 [1,37]. It has been shown that the G+C content of the genome of the virophage YSLV 5 is 51.1%, which is typical for algal host cell virophages and significantly higher than the average value of this parameter for protozoan host cell virophages, as well as YSLV 6–7, which contain 26.7% and 27.3% G+C, respectively. This may indicate a host cell range unique to the virophage YSLV 5 [37]. It has been shown that 11 of the 32 genes of the YSLV 5 virophage are homologous to the genes of the other YSLV virophages, except that g06 and g11 are also very similar to g12 of the Sputnik virophage and g20 of the ALM virophage, respectively [37]. It was also recorded that virophage YSLV 7 has 11 genes homologous to the genes of the described virophages out of 32 genes, seven of which are most similar to the genes of Virophages YSLV 1–6 [37]. In addition, in virophages YSLV 5, YSLV 6, and YSLV 7 identified five conserved core genes specific to the described virophages encoding the putative DNA helicase (HEL), the packaging ATPase, the cysteine protease, and the MCP and mCP proteins [37]. Furthermore, it has been shown that, in addition to the helicase gene, the other gene products of these virophages show an amino acid similarity of 42–62% with their counterparts in Virophages YSLV 1–4. This would suggest their early evolutionary divergence between them and indicates that Virophages YSLV 5–7 and YSLV 1–4 are somewhat distinct, although certainly more closely related to each other, relative to other known virophages [37].

### 3.2. Virophage Zamilon 2

The Virophage Zamilon 2 was described in 2015 as homologous to the virophage Zamilon, which was recorded in the metagenome of a bioreactor of poplar wood material (sawdust) in New York State, USA, already in 2012 [1,2,11,17,37,43]. This virophage is assumed to probably colonize unknown giant viruses parasitizing amoebae of the genus Acanthamoeba [2,55]. Most likely similar to the Zamilon virophage, it has an icosahedral capsid [1,38,43], but its dsDNA genome consists of only 6716 bp, of which 392 bp are identical to that of the Zamilon virophage, corresponding to a 39% similarity between these virophages [1,2,38]. Zamilon Virophage 2 has 15 genes homologous to genes found in Zamilon virophage, which have 78–99% amino acid similarity and 81–96% nucleotide identity, indicating that they are sister virophages [1,25,38]. Homologous proteins in Zamilon 2 and Zamilon virophages have also been shown to be MCP protein, DNA replication protein, and ATPase-packaging protein [25,38].

### 3.3. Virophages from Lake Mendota and Trout Bog

These virophages were already recorded in 2009 and 2012 but were only described in 2017, presenting them as 25 new “non-breeding” virophages, including 17, presumably, complete genomes [25,39]. They were found in the urban freshwater eutrophic Lake Mendota and the acidic Trout Bog in Wisconsin, USA [39]. They differ from the virophages Sputnik and Mavirus, in that their genome is based on conidial circularity or inverted terminal repeats, and their predicted complete dsDNA genomes consist of 13.8–25.8 kbp, and from 13 to 25 genes [25]. Only four of these, out of 13–25 genes, were shown to be likely “core”, as they were familiar to almost 25 new virophages described, although of the 25 virophages shown, two virophages are characterized by the absence of one of these four mentioned genes and form linear partial genomes lacking the region encoding the DNA packaging protein (TBE_1002136—Trout Bog Epilimnion1002136) [39]. In contrast, of the remaining four genes, three genes form the hybrid genome of the RPV virophage found in the rumen of sheep, characterized by the absence of the mCP protein [39]. In addition, two other genes, out of the four mentioned, which were identified as core in their genome, were shown to encode a primase-helicase and Zn ribbon domain protein of unknown function and were present in 68% and 80%, respectively, in the genomes of these virophages. Hence, they were classified as “near-core” genes [39]. Of the 25 new “non-culturable” virophages described, 16 encode a tyrosine recombinase integrase similar to that found in the OLV virophage, indicating that these virophages may integrate into the genome of eukaryotic hosts. In addition, seven of these 16 virophages contained a putative DNA polymerase α (Polα) family, also found in Sputnik virophage, while two encode Polβ described in Mavirus virophage [39]. It should be added that phylogenetic studies on these 25 new “uncultured” virophages have shown that the genes encoding the Polβ proteins of the Mavirus virophage, evolutionarily related to a group of mobile eukaryotic genetic elements termed Maverick/Polinton, are highly related to sequences of bacterial viruses of the *Tectiviridae* family and *archaea* viruses [39]. It should be added that in Trout Bog, none of the virophages shown in the waters of Lake Mendota were detected and vice versa, confirming that these two contrasting freshwater ecosystems represent distinct virophage communities [39].

### 3.4. Virophages DSLV 2–8 (Dishui Lake Virophages)

Virophages DSLV 2–8 were described in 2018 as seven complete genomes already recorded in 2016 in the freshwater of Dishui Lake, Shanghai, China, which reside on various giant viruses of the family *Phycodnaviridae* infecting unspecified freshwater algae, although possibly also on giant viruses of the genus *Mimivirus* infecting amoebae [25,40]. Their sequence lengths are for DSLVs 2–8, 31,238; 31,512; 30,873; 26,593; 28,714; 29,961; and 26,605 bp, respectively, and they contain between 32.3 and 45.2% G+C, which is similar to the G+C content of PLVs in which this value ranges from 30 to 39% [40]. It has been recorded that Virophages DSLV 2–8 are similar to the virophage PGVV, in which a relatively low G+C content of 36% has also been observed, which is related to the giant virus PgV–16T infecting marine algae of the genus *Phaeocystis* [40]. It should be added that in most of the described virophages involved in the CVV system, for example, the virophage CVV-SW01 parasitizing algal-infecting giant viruses, the G+C content only oscillates by approximately 30%, which distinguishes them from the virophages DSLV 2–8, which mainly infect algal-infecting giant viruses [40]. Virophages DSLV 2–8 encode 38 (DSLV 2), 31 (DSLV 3), 32 (DSLV 4), 25 (DSLV 5), 29 (DSLV 6), 30 (DSLV 7), and 25 (DSLV 8) genes s, respectively, indicating that DSLV 2 virophage, among DSLV virophages, as well as other described virophages, contains the most genes [40]. The DSLV 2–8 virophages genome is a circular dsDNA, except for DSLV3 virophage, whose genome contains palindromic repeats in the asymmetric position of the two ends of the genome, which is reminiscent of the linear genomes found in *Mimivirus* giant viruses [40]. DSLV 2–8 virophages are closely related and are similar to virophages isolated from lake waters, especially YSLV 3–4 and Lake Mendota virophages [40]. Among the DSLV 2–8 virophages, four core genes were identified, i.e., the genes encoding packaging ATPase, cysteine protease, MCP protein, and mCP protein, and one putative DNA helicase gene not found in DSLV 2 and DSLV 3 virophages [40]. Three pairs of virophages are most closely related: DSLV 1/7, DSLV 4/6, and DSLV 5/8, which show greater relatedness than has been recorded in Virophages DSLV 2 and DSLV 3 [40]. A relatively weak similarity of DSLV 2–8 virophages with virophages parasitizing giant viruses living on protozoa was observed, demonstrating their different evolution. DSLV 2–8 virophages are closely related to giant viruses of large algae, e.g., DSLPV 4 (Dishui Lake phycodnaviruses 4) and DSLLAV 1 (Dishui Lake giant alga virus) [40], in which a nonhomologous system similar to CRISPR–Cas in bacteria has been described (DSLLAV 1) and appears to protect this giant virus from the destructive effects of DSLV 5 and DSLV 8 virophages [40]. This arrangement suggests that in Lake Dishui, there is probably also a tripartite CVV system, that is, virophage—giant virus—algae [40], homologous to that also recorded for the virophage CVV–SW01 [23].

### 3.5. Virophages LCV (Loki’s Castle Virophages)

Researching the metagenome of marine sediment from the Loki Castle hydrothermal vent area—named after the Norse God of Fire located on the Mid-Atlantic Ridge in the Arctic Ocean—two Sputnik-like virophages, LCV 1 and LCV 2, were described in 2019 without specifying their genome and shape and capsid symmetry [16]. A phylogenetic study of their MCP protein showed that although they form a distinct branch within the “Sputnik-like” group of virophages, they are parasites of giant viruses of the genus *Mimivirus*, for which no host cell has been identified [16]. In addition to their main MCP protein, they encode an mCP protein, an ATP-packing ATP-ase, and a cysteine protease. Although they lack the gene encoding the primase-helicase fusion protein, each of their genes encodes a distinct helicase, distinguishing them from the Sputnik virophage [16]. It is interesting to note that LCV 1 and LCV 2 virophages also contain a conserved A+T-rich motif under each gene and probably correspond to the late promoter of their hosts cells, as is the case for virophages carrying the late promoters of *Mimivirus* giant viruses [1,14,16,25]. It should also be added that as the genomes of the two putative *Klosneuviruses* (LCMiAC01 and LCMiAC02) do not contain equivalent LCV virophage promoters, it has been suggested that it is possible that their host cell could be not only *Mimivirus* giant viruses but also *Pitoviruses*, for which, as for *Mimiviruses*, no host cell has been identified [16].

### 3.6. Virophage Guarani

This virophage was described in 2019 in freshwater samples collected in the Pampulha lagoon in Belo Horizonte, Brazil, and, like the virophage Sputnik 3, it may be “free” of its specific giant virus [1,2,14,17,25]. This virophage was named Guarani because the South American Guarani tribes live near where it was found [14]. The virophage has been shown to have a long replication cycle during infection with giant viruses of the *Mimiviridae* family from the A, B, and C lineages [25,27], during which it acquires a unique deletion mutation pathway in g08, which is a strong resemblance to the giant virus *Tupanovirus* [14]. Evaluating the replication of the Guarani virophage cultured on the *ACMV* giant virus in the amoeba *A. castellanii*, it was shown that its long replication cycle is linked to its delayed replication associated with the utilization of the late promoter of this giant virus, which starts at the latest stage and extends to the final stages of its morphogenesis process [1,13,14,25]. The Guarani virophage has an icosahedral capsid symmetry of 50–60 nm in diameter, and its double-stranded DNA is a circular genome with a length of 18,967 bp and a G+C content of 26.8% [33]. Its genome consists of 22 genes ranging from 342 to 2340 bp in length and is very similar to that of the virophage Sputnik [1,2,14,17,25], although the exceptions are its g19 and g12 [1,2,14,25]. ORF 19 is related to the Zamilon virophage, while g12 does not match any sequence described thus far in virophages because the G+C content of this gene is only 18%, which distinguishes this gene from the G+C content of the genes of other protozoan host cell virophages, which have an average G+C content of approximately 30% [1,2,14,25]. It may suggest that g12 of the Guarani virophage was introduced into its genome by horizontal gene transfer because the other 20 genes, out of 22 genes, show high similarity with the Sputnik gene [14]. BLASTp analysis showed that g08 and g09 of this viroid encoded a collagen-like protein, g13 is presumably an integrase, and g20 is presumably a transposase, although some of them also encode conserved core proteins of this viroid [14]. g01 and g22 encode an MCP protein, g10 and g21 encode an mCP protein, g05 encodes a DNA packaging ATPase, g11 encodes a cysteine protease, and g16 encodes a DNA replication protein [14]. In addition, g17 has been shown to encode an unidentified protein that shows high amino acid similarity to the protein encoded by Sputnik’s g14 (98% overlap and 92% identity), as well as the Zn ribbon protein domain [14]. It is hypothesized that the likely orthologues of g18 of this virophage also encode a transmembrane protein containing a putative conserved domain from cytochrome C oxidase subunit II [14], which has moderate homology to g15 of the Sputnik virophage (68% identity) and g01 of the Zamilon virophage (45% identity) [14]. g19 of the Guarani virophage is distinct from the Sputnik virophage but is homologous to g03 of the Zamilon virophage (41% identity) and encodes a protein of unknown function [14]. g20 of this virophage may be similar to its g17, as 96% of the identity in their amino acids has been demonstrated [14]. It has also been indicated that the other genes of the Guarani viroid encode unidentified proteins [14]. Because the genome architecture of the Guarani virophage is very similar to that of the Sputnik and Zamilon virophages, they are assumed to share a common origin [14]. It has also been proven that the Guarani virophage, by infecting giant viruses of the *Mimiviridae* family Lineages A, B, and C, affects their replication and infectivity, resulting in a decrease in their abundance by up to 90% and consequently increasing the survival of the amoebae and protists on which these giant viruses parasitize [14,48].

### 3.7. Virophage Sisivirophage

This virophage was recorded in 2019 in Tunisia but has not been fully described [1]. It has only been reported that its genome is very different from known virophages and is a DNA material whose shape has not been determined, and the symmetry of its capsid has not been described [1]. A phylogenetic tree based on the MCP protein of this virophage showed that its closest homologue is virophages obtained from metagenomic data from the waters of Lake Mendota, USA [1].

### 3.8. Virophages from Gossenköllesee Lake

In the form of 32 genomes, these virophages were described in 2021 in the waters of the oligotrophic alpine lake Gossenköllesee in 2021 in Austria, although they had already been recorded in 2017 and 2018 [41]. These virophages have a circular dsDNA genome and a characteristic gene encoding the MCP protein [18,41]. By studying these 32 virophages [41], the homology of their genomes to the genomes of other virophages has been demonstrated, in particular to the genes of the packaging ATPase, the mCP protein and the MCP protein, and which genes also show some similarity to the MCP protein genes of giant viruses [41]. In these 32 virophages, inverted terminal repeats (TIR), frequently recorded in polintons, PLVs, and virophages, were detected in their genomes [41]. Furthermore, demonstrating TIR sequences in the genomes of these 32 virophages has determined that they are complete, although it has not been fully documented whether they are free viral particles or forms of proviruses [41].

## 4. Hypothetical Use of Virophages in Practice

The effect of virophages on the human body is not yet known. However, the effect of *Mimivirus* giant viruses on humans has been demonstrated, as they have been registered, among others, as an infectious agent in patients with CAP (community-acquired pneumonia), and antibodies to collagen encoded by these viruses are involved in human rheumatoid arthritis [17,20,43,48]. On the other hand, it has been recorded through virophages that there may be a novel pathway for inducing immune responses in mammals [48]. It has also been indicated [17,43] that virophages of giant viruses can be used as vaccines, as they could theoretically be used as potential prophylactic elements in patients affected by CAP. It is assumed that genetically modified virophages could also be used against the SARS-CoV-2 virus infection [17]. In these considerations, one looks [17,43] at the possibility of constructing a virophage almost genetically identical to the SARS-CoV-2 virus but with the gene encoding the RNA polymerase excluded and the reverse transcriptase (RTase) gene added, which would produce a replicase–transcriptase complex containing RTase. However, no replicase, as the absence of replicase (RNA-dependent RNA polymerase) makes the virophage compete for it with a “helper virus;” in this case, the coronavirus.

Moreover, the replication and expression of the virophage genome leads to reverse transcription of viral and virophage RNA [17,43], preventing transcription by reverse transcription of further viral RNA replication or translation. Such a modified virophage would inactivate the coronavirus and, at the same time, prevent it from damaging cells in the human body [17,29]. Performing such an experiment, despite the high infection specificity of virophages and their instability outside the host cell, is possible [17,43]. It should be added that the incentive to use virophages in such a procedure is the potential use of the fact that virophages are unable to replicate independently of giant viruses for vector construction and gene insertion. This is of great importance in the development of targeted therapy, especially as virophages have a small size and a relatively simple genome.

## Figures and Tables

**Table 1:** Described virophages.

**Virophages with the Described “Host” and Its Host Cell**							
**L.P.**	**Name of the Virophage**	**Name of the Giant Virus and (or) Its Family and Genus **	**Eucaryotic Host of the Giant Virus**	**Year of Statement and/or Description**	**Item of Literature ^x^**	**Genome Size**	**Accession ID**
1.	Sputnik	*Mamavirus ACMV*	*Acanthamoeba* (*A.*) *castellani*	2008	[26,27]	18,343	EU606015
		*Mimivirus APMV*	*A. polyphaga*		[26,27]		EU606015
2.	Sputnik 2	*Lentille virus*	*A. polyphaga*	2012	[26]	18,338	JN603369.1
3.	Sputnik 3	*Mimiviridae* mainly of the C lineage or virophage is “free” of the giant virus	*A. polyphaga* for viruses of the genus *Mimivirus*	2013	[4]	18,338	JN603370.1
4.	Sputnik argentum	*Mimivirus argentum*	Probably genus amoebas *A. castellani*	2022	[28]	18,800	34293
5.	*Mavirus*	*Cafeteria* *roenbergensis virus*	Flagellate *Cafeteria roenbergensis*	2010	[17]	19,063	GCF_000890715
6.	OLV (Organic Lake Virophage)	Viruses of the *Phycodnaviridae* family	Phototrophic marine algae—unnamed	2011	[29]	26,421	HQ704801
7.	RNV (Rio Negro Virophage)	Samba virus	*A. castellanii*	2011	[30]	18,145	MG676470
8.	PGVV (Phaeocystis Globosa Virus Virophage)	PgV-16T virus (*Phaeocystis* globose virus)	Algae of the genus *Phaeocystis*	2013	[21,31]	19,527	KC662249–KC662250
9.	ALM (Ace Lake Mavirus)	Probably viruses from the *Mimiviridae* family	Protozoa unspecified	2013	[32]	17,767	No data available
10.	YSLV 1 (Yellowstone Lake Virophages 1)	Probably viruses from the *Phycodnaviridae* or *Mimiviridae* families	Unnamed algae or unspecified amoebas	2013	[22,32]	27,849	KC556924
11.	YSLV 2					23,184	KC556925
12.	YSLV 3					27,050	KC556926
13.	YSLV 4					28,306	KC556922
14.	Zamilon	*Mont1* virus	*A. polyphaga*	2014	[33]	17,276	JX484142
15.	RVP (Rumen virophage)	Probably viruses from the *Mimiviridae* family	Indeterminate eukaryotic host—protists	2015	[27]	26,209	No data available
16.	DSLV 1 (Dishui Lake Virophage 1)	Probably viruses from the family *Phycodnaviridae*	Freshwater algae, unspecified	2016	[34]	28,788	No data available
17.	QLV (Qinghai Lake Virophage)	Probably viruses from the family *Phycodnaviridae*	Freshwater algae, unspecified	2016	[35]	23,379	KJ854379.1
18.	Platanovirus saccamoebae virophage “Comedo”	KSLT virus probably belongs to the *Mimiviridae* family	*Saccamoeba lacustris*	2018	[5]	No data available	No data available
19.	CpV-PLV Curly	CpV-BQ2 virus	Fresh water algae—*Chrysochromulina parva*	2019	[36]	22,761	MH919296
20.	CpV-PLV Moe					21,750	MH919297
21.	CpV-PLV Larry					22,879	MH920636
22.	CVV-SW01 (Chlorella virus virophage)	*Chlorella* virus -CV-XW01	Freshwater algae of the genus *Chlorella*	2022	[23]	24,744	OL828819
	Virophages with an undescribed or probable “host” and their possible host cell						
1.	YSLV 5	Undefined	Undefined	2013	[37]	29,767	KM502589
2.	YSLV 6					24,837	KM502590
3.	YSLV 7					23,193	KM502591
4.	Zamilon 2	Probably giant viruses—unspecified	Probably amoebas of the genus *Acanthamoeba*	2015	[38]	6716	No data available
5.	Virophages from Lake Mendota and Trout Bog fen	Undefined	Undefined	2017	[39]	13,800–25,800	No data available
6.	DSLV 2 (Dishui Lake virophages)	Probably giant viruses of the family *Phycodnaviridae* and possible viruses of the genus *Mimivirus*	Freshwater algae unspecified and/or amoebas	2018	[40]	31,238	MN940570
7.	DSLV 3 (Dishui Lake virophages)					31,512	MN940572
8.	DSLV 4 (Dishui Lake virophages)					30,873	MN940571
9.	DSLV 5 (Dishui Lake virophages)					26,593	MN940574
10.	DSLV 6 (Dishui Lake virophages)	Probably giant viruses of the family *Phycodnaviridae* and possible viruses of the genus *Mimivirus*	Freshwater algae unspecified and/or amoebas	2018	[40]	28,714	MN940573
11.	DSLV 7 (Dishui Lake virophages)					29,961	MN940576
12.	DSLV 8 (Dishui Lake virophages)					26,605	MN940575
13.	LCV 1 (Loki’s Castle Virophage 1)	Viruses of the genus *Mimivirus*, and maybe *Pitoviruses*	Undefined	2019	[16]	No data available	No data available
14.	LCV 2 (Loki’s Castle Virophage 2)						No data available
15.	Guarani	The virophage is “free” of the giant virus, or they are viruses from the *Mimiviridae* family	For viruses of the genus *Mimivirus*, possibly unnamed amoebas and/or marine protists	2019	[14]	18,967	LS999520
16.	Sisivirophage	Undefined	Undefined	2019	[1]	no data available	No data available
17.	Virophages from Lake Gossenköllesee	Undefined	Undefined	2021	[41]	no data available	No data available

Explanations: ^x^—additional references in the text.
